# Stable isotope analyses of carbon and nitrogen in hair keratin of suspected man-eating wolves from 1880s

**DOI:** 10.1038/s41598-024-55521-8

**Published:** 2024-02-28

**Authors:** Juho-Antti Junno, Tiina Väre, Jouni Tikkanen, Matti T. Heino, Markku Niskanen, Iiro Kakko, Johanna Honka, Titta Kallio-Seppä, Laura Kvist, Jenni Harmoinen, Jouni Aspi

**Affiliations:** 1 Archaeology, University of Oulu, Oulu, Finland; 2 Anatomy, University of Oulu, Oulu, Finland; 3 Laboratory of Chronology, Finnish Museum of Natural History, University of Helsinki, Helsinki, Finland; 4 Ecology and Genetics Research Unit, University of Oulu, Oulu, Finland; 5 Department of Forensic Medicine, University of Helsinki, Helsinki, Finland; 6 The Museum of Torne Valley, Tornio, Finland

**Keywords:** Animal behaviour, Behavioural ecology

## Abstract

The so-called man-eating wolves of Turku, a pack of three wolves, reportedly killed 22 children in South-Western Finland in 1880–1881. Enormous efforts were carried out to eradicate them. In January 1882 the last remaining wolf was killed. Since then, there has been considerable debate regarding the validity and extent of the man-eating behaviour. This study aims to clarify whether man-eating behaviour can be observed from the remains of these wolves. One of the wolves was mounted in 1882 and is on display at St. Olaf’s school in Turku, enabling us to collect hair keratin samples. Additionally, hair keratin was collected from two other suspected man-eaters. We analysed carbon (δ^13^C) and nitrogen (δ^15^N) stable isotope values to study the wolf’s diet during the last months of its life. Samples from seven temporally concurrent wolves were used to construct reference values. Our analyses indicated that δ^15^N values of suspected man-eaters were relatively low compared to the reference sample. We could not detect clear trends in isotope ratios associated with potential man-eating behavior. We believe that this lack of distinctive patterns can be explained by the relatively minor role that man-eating played in their overall diet.

## Introduction

**Figure 1:**
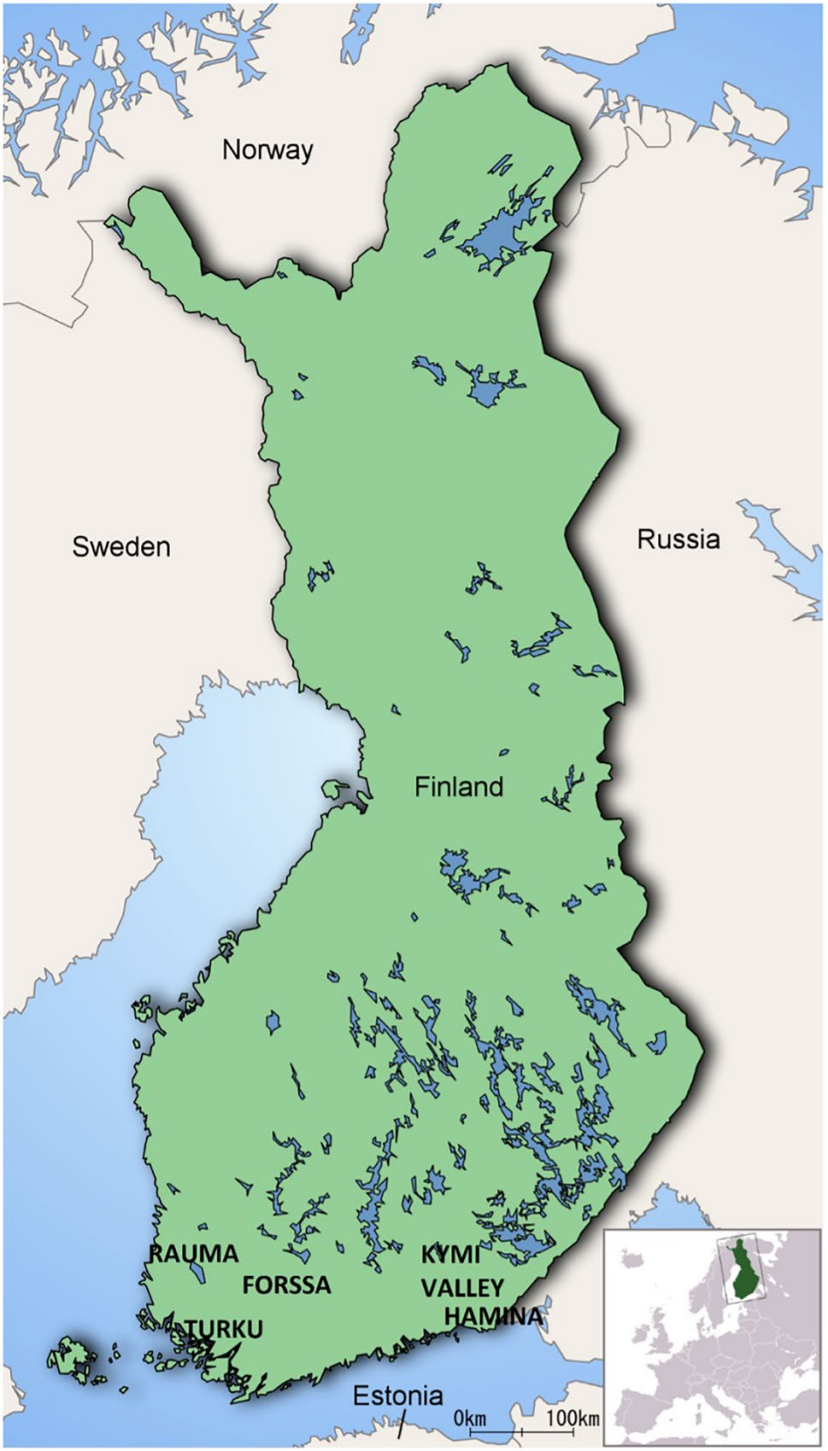
A modern map of Finland presenting the relevant sites. Man-eating occurred in Turku region and our reference wolf sample are mainly from Kymi Valley and Hamina. Our archeological reference human samples originated from Rauma. Map: Public domain, Wikimedia Commons, modified by Tiina Väre.

In 1880 and 1881 the so-called man-eating wolves of Turku reportedly killed 22 children^1,2^ in Southwestern Finland (Fig. 1). The first incident occurred on 18 January 1880, in Vellua village, 50 km northwest of Turku, the former capital of Finland. Two wolves were spotted from a neighboring house crossing a potato field and carrying something. That something turned out to be a boy named Karl Johan Hörnberg, later found dead and partly eaten in a nearby forest^3^.

During the following 22 months, the wolves were reported to have attacked 26 other children and one adult in small countryside villages north and northeast of Turku. In total, 22 children aged 2–10 years died^1,2^. Most of them were eaten at least partially. Large-scale wolf hunts were organized during the summers of 1880 and 1881 to eradicate this pack. Bounties placed by both the Senate and the county councils lured professional hunters to the area in the wintertime. Poisoned carcasses, trapping, and killing of the cubs during the spring thinned out the local wolf population. However, it took 2 years and even a group of *lukashi*—professional wolf hunters from Russia—to track and kill the alpha pair of the man-eating pack in January 1882^1,2,4^. The cubs of the pack were killed during the summers of 1880 and 1881, with only one cub surviving until the end of October 1881^1,5^.

The alpha female of the pack, killed on 3 January 1882^6^, was found to be in poor condition, with worn-out teeth and a shaggy, yellow-brown fur^1^. The fur of the female was preserved but is believed to be currently missing. The alpha male was killed on 14 January 1882 by a son of a tenant farmer, Matti Hillberg, who sold the wolf’s hide for mounting^7^. The mounted wolf was later donated to a local school in Turku, where it is still preserved^2^. By the time of the killing, the male was reportedly tired, lame, and walking on three legs, apparently because he had been shot at the hind leg during the hunt on 3 January 1882^1^. The exact size of the wolf pack engaging in man-eating behavior is, however, uncertain. The man-eating pack could have consisted of just the alpha pair, the alpha pair and their cub(s), or possibly also other wolves in the area.

## Wolf diets and potential man-eating behavior

In Finland, European moose (*Alces alces alces*) is the main source of nutrition of wolves^8,9^. However, wolves are opportunistic if their preferred prey is scarce or unavailable^10,11^. In the late nineteenth century, extensive hunting had seriously reduced the moose population in southern Finland^12^, and it is likely that wolves of Turku hunted small prey animals and raided local farms for nutrition. For example, in 1878, 12 pigs, 2455 sheep, 472 cows, and 158 horses were lost to predators in the Turku region^2^. Limited prey availability is observed to be one of the main factors associated with man-eating behaviour in carnivores^13^. It is thus not difficult to understand the idea that in the absence of prey, the wolves would have resorted to hunting children.

## Stable isotope analyses

Each tissue stores the stable isotope composition representing the diet conditions during its formation^14,15^. In this paper, the ratios of the stable isotopes of carbon (^13^C/^12^C) and nitrogen (^15^N/^14^N) in body hair keratin have been used to reflect the dietary habits of wolves during the period of hair growth^16^.

Wolves molt once a year, during the late spring (May–June), when they shed their warm winter fur. In the autumn, they grow a thick underfur for the cold winter months. Several factors are associated with the body hair growth in wolves. Hair growth rate vary among individuals and among different body parts of the same animal^17^. The growth rate of body hair of captive wolves was found to be 1.0 ± 0.07 mm/day in the summer and 0.63 ± 0.23 mm/day in the autumn^16^.

The isotopic compositions of the samples are measured in parts per thousand (‰) and are expressed with a delta (δ) notation, indicating the relative difference of isotope ratios between the sample and a standard (Vienna Peedee Belemnite for carbon and AIR for nitrogen). Earlier studies have demonstrated that the remains of man-eating carnivores can be utilized to study such behavior in detail. For example, stable isotope analyses were used with famous Tsavo lions to reveal the actual extent of their man-eating behavior^18^. The feasibility of determining a diet from partitioned hair samples has been studied in a controlled feeding study of captive wolves^16^.

In healthy, well-nourished individuals the ratio of nitrogen stable isotopes (ẟ^15^N) of most tissues can be expected to reflect the consumption of animal proteins^14,15,19,20^. Additionally, it can be used to determine the trophic position of the organism by comparing the values to local faunal data, considering trophic enrichment factors of 3–5‰^21^.

The ratio of carbon stable isotopes (δ^13^C) allows studying whether the analysed organism consumed plants using the C_3_ or C_4_ photosynthetic pathway or other organisms consuming C_3_- or C_4_-plants and to trace marine influences in the diet^22–25^. In brackish water environments, such as the Baltic Sea, as in freshwater environments, the carbon values likely resemble those of terrestrial C_3_ environments^26^. Thus, locally distinguishing between marine and terrestrial environments based on δ^13^C values is not possible. On the other hand, in addition to ẟ^15^N values, starvation may affect δ^13^C values^27–30^. Another, more geographically relevant factor possibly shaping the δ^13^C values is the canopy effect causing the ground level plants to be depleted in ^13^C compared to those higher in the canopies^31^. Also lichen-eating reindeers as a part of the trophic web can slightly elevate the δ^13^C values^32^, but reindeer are not present in the southern region of Finland, where the wolves of this study originate from.

In this study, we conducted experiments to determine whether man-eating could be observed from stable isotope values in hair samples taken from the alpha male, the St. Olaf’s school wolf, as well as two other wolves, potentially other individuals from the man-eating wolf pack. The δ^13^C and δ^15^N values in body hair keratin reflect dietary habits during the last couple of months prior to the death. Our hypothesis was that comparing the stable isotope values of the potential man-eater’s hair keratin to those measured in a concurrent reference group would allow us to further assess the man-eating behaviour. Significant consumption of human tissue should produce statistically different δ^15^N values in the hair keratin compared to our reference samples.

## Results

### The volume of human biomass consumed

The amount of consumed human tissue was approximated from the weights of the children by their age. The month starting from June 15th onwards included four confirmed human kills by the wolves. A 6-year-old boy was half-eaten (equating 8–12 kg of human tissue)^33^ as well as parts of an 8-year-old boy (~ 10 kg)^34^, a 2-year-old boy almost entirely (~ 10 kg)^35^ and parts of an 8-year-old girl (~ 8 kg)^36^.

During the month from August 15th onwards, only two kills could be confirmed. A 4-year-old boy was killed and concealed, but not eaten^37^ and a 9-year-old boy was partially eaten (~ 9 kg)^38^. The next month, starting from September 15th, included just one kill. An 8-year-old boy was almost completely eaten (~ 22 kg)^39^.

The month from October 15th onwards included two kills. A 7-year-old girl was killed and dragged away, but wolves were disturbed, and they could only eat parts of the girl (~ 9 kg)^40^. Moreover, a 5-year-old boy was killed on November 7th but only partially eaten, potentially mainly organs (~ 6 kg)^41^. This was the last human kill by the wolves, and there was approximately a two-month gap in human kills until the alfa pair was killed. During their last six months of existence, we estimated that the man-eating wolf pack of Turku could have been able to obtain approximately 85 kg of human biomass in their diet.

### Stable isotopes

The keratin C:N atomic ratios for two wolves in the control group were outside the range (3.0–3.8) for keratin suggested by O’Connell and Hedges for human hair keratin^20^. However, unlike for collagen, the atomic C:N ratio of keratin is not considered as a strong indicator of preservation which is why these two samples were chosen to be included in the analyses. All other hair samples were within the suggested range for hair keratin (Table 1).

**Table 1:** Stable isotope analyses of carbon and nitrogen in hair keratin of the reference wolf samples, suspected man-eating wolves (359 and Forssa) and the average values for the man-eating wolf from St. Olaf’s school Turku from the sequentially cut hairs. The keratin values were modified to correspond the more frequently used collagen values according to the suggestions of O’Connell and Hedges^20^ by adding + 0.5‰ to the δ^13^C values and 1‰ to the δ^15^N values. Additionally, the δ^13^C values were adjusted for the SUESS effect according to the table presented by McCarroll and Loader^54^.

	δ^13^C (‰)	δ^15^N (‰)	Mod. δ^13^C (‰)	Mod. δ^15^N‰	C%	N%	C/N atomic
Reference group							
Wolf 400	− 21.9	9.4	− 21.3	10.4	42.2	14.1	3.5
Hunting museum 1	− 21.0	8.8	− 20.4	9.8	42.6	14.2	3.5
Hunting museum 2	− 19.6	10.7	− 19.0	11.7	42.6	14.2	3.5
Hunting museum 3	− 19.5	10.7	− 18.9	11.7	39.7	16.8	2.8*
Hunting museum 4	− 20.4	10.3	− 19.8	11.3	41.9	13.9	3.5
Hunting museum 5	− 17.0	8.3	− 17.1	9.3	40.8	18.3	2.6*
Hunting museum 6	− 20.0	8.2	− 19.4	9.2	41.3	13.8	3.5
Potential man-eaters							
St. Olaf’s school wolf mean	− 21.9	7.8	− 21.2	8.8			
Wolf 359	− 22.3	8.3	− 21.7	9.3	38.9	13.8	3.3
Forssa	− 21.9	7.8	− 21.3	8.8	41.2	13.4	3.6

*Atomic C:N ratios beyond those suggested as quality criteria for well-preserved keratin/collagen.

**Figure 2:**
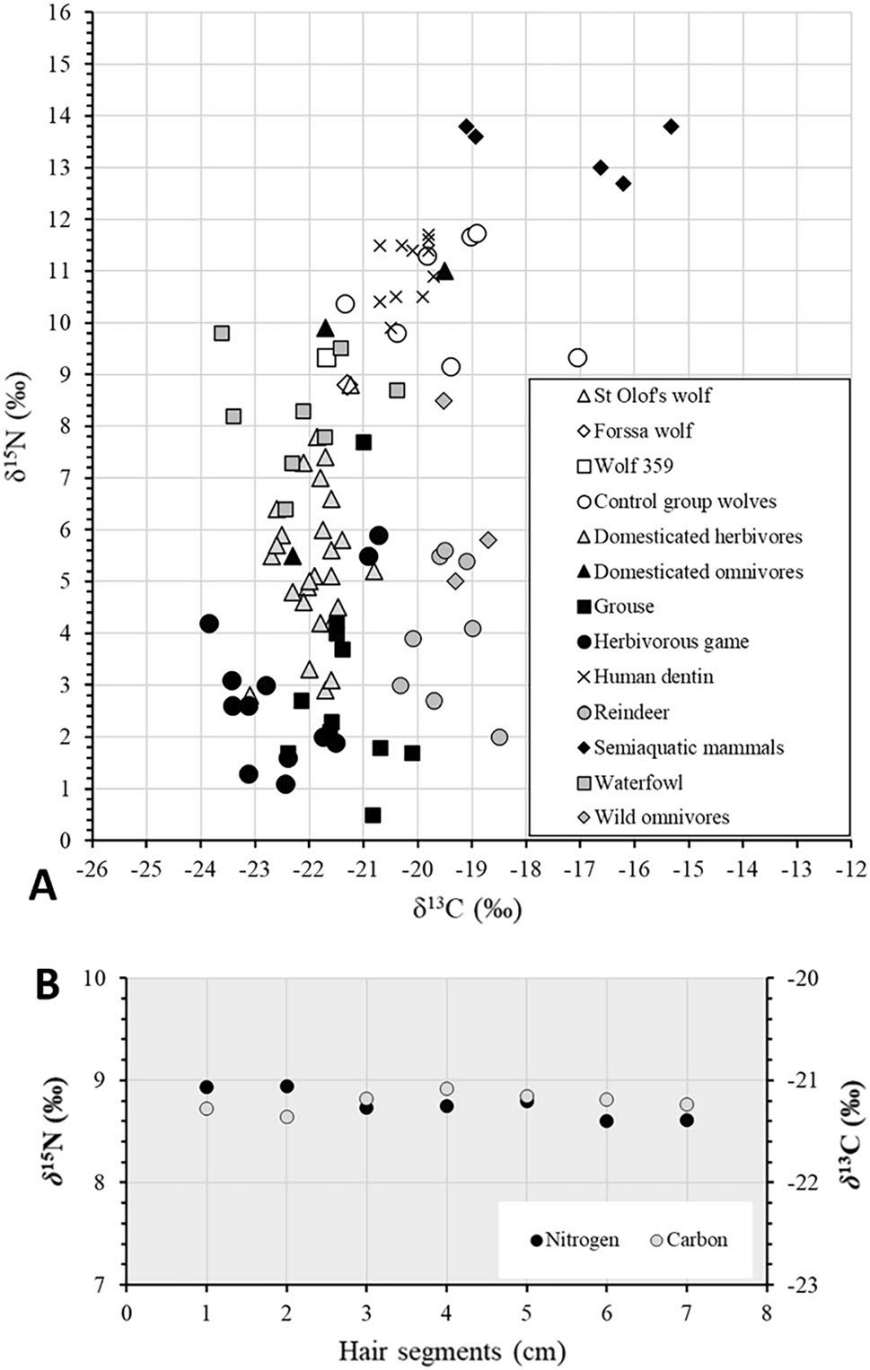
δ^13^C and δ^15^N values of the wolves and the reference fauna. (**A**) The modified keratin δ^13^C and δ^15^N values of the reference wolves, suspected man-eating wolves (359 and Forssa) and the average value for the man-eating wolf from St. Olaf school from the sequentially cut hairs. The faunal background bone/dentin collagen isotopic data is from archaeological (Middle Ages to Early modernity) or ecological (pre-1950) animal remains from Finland^49,55–58^) The human dentin collagen isotopic data is from the early nineteenth century population of Rauma located near Turku^44^. (**B**) The δ^13^C and δ^15^N isotope values in the sequential hair samples of the St Olof school’s wolf. The variation is 0.2‰ for δ^13^C and 0.3‰ for δ^15^N while the analytical error for both is 0.1‰.

Due to the small sample sizes, the nonparametric Mann–Whitney test was selected to analyze whether the suspected man-eating wolves presented different stable isotope values than the wolves in the reference group. The δ^15^N value in the keratin of the St Olaf school’s wolf and Forssa wolf were the lowest in comparison to the reference group (Table 1; Fig. 2A). According to this test, the keratin values of the suspected man-eaters (n = 3) statistically deviate from those of the reference group (n = 7) (δ^13^C p 0.033; δ^15^N p 0.033). This suggests that the isotopic δ^13^C and δ^15^N composition of the diet consumed by the suspected man-eaters during the hair development was dissimilar to the reference group containing a clearly smaller portion of protein from higher trophic positions. This is not in line with the consummation of flesh of humans who typically occupy higher trophic positions.

Both the δ^13^C and δ^15^N values of the St Olaf school’s wolf showed very little variation across the hair shaft (Table 2; Fig. 2B). The increase in nitrogen levels during the entire period was only 0.3‰, and the carbon values among sequences varied even less, with the variation remaining at 0.2‰ which could be nearly completely explained by analytical imprecision.

**Table 2:** The isotope values of hair strands of the St. Olaf’s school wolf analysed in 1 cm sequences from the root towards the ends (1–7).

Sequence	δ^13^C	δ^15^N	Mod. δ^13^C	Mod. δ^15^N	N%	C%	C/N atom
1	− 21.9	7.9	− 21.3	8.9	13.92	40.73	3.4
2	− 22.0	7.9	− 21.4	8.9	13.79	40.63	3.4
3	− 21.8	7.7	− 21.2	8.7	13.77	40.67	3.4
4	− 21.7	7.8	− 21.1	8.8	13.72	40.65	3.5
5	− 21.8	7.8	− 21.2	8.8	13.63	40.45	3.5
6	− 21.8	7.6	− 21.2	8.6	13.65	40.43	3.5
7	− 21.9	7.6	− 21.2	8.6	13.36	40.52	3.5

In addition to hair samples, isotopic data was attempted to obtain from a bone of the taxidermized St. Olaf’s school wolf. The atomic C:N ratio (4.1) of the bone collagen sample fell outside the accepted range of 2.9–3.6 suggested by DeNiro^42^, indicating dissatisfactory collagen quality. Thus, the obtained values (δ^13^C − 22.8‰; δ^15^N 8.2‰) were not included in the analysis. This is unfortunate as they would have reflected the long-term average diet of the wolf and could have been used as the baseline with which to compare the keratin values.

## Discussion

The relatively low keratin δ^15^N values of the St. Olaf’s school’s wolf are not well aligned with the suspected man-eating behaviour, which should have elevated the trophic level of the wolf. The lack of variation in the values across the hair shaft implies a steady diet during the period of the hair growth. The δ^15^N values of the two other suspected, but less well documented man-eaters, wolf 359 and Forssa wolf, were somewhat lower in relation to the reference group.

An adult wolf with a body weight of 35 kg is estimated to require 3.25 kg of food per day. There is a significant variation in observed daily food consumption levels; depending on food abundance, the consumed amount could be even more than 10 kg/day^43^. The wolf pack under study consisted of an adult alfa pair, to which an offspring was added during the summer of 1881. Based on the daily food requirements, it is obvious that an estimated human biomass of 85 kg can explain just part of the diet of these wolves. It could comprise roughly 10–15% of their diet during the months of most frequent kills as the total food requirement for the pack should have been at least 200 kg/month.

Our results cannot conclusively support or rule out the man-eating behavior of the studied wolves. While the hair keratin ^15^N values were depleted relative to the human dentine values suggest that human flesh likely did not play a significant role in their diets during their last months, small portions would not necessarily have elevated the δ^15^N values, particularly if the diet primarily consisted of low ^15^N content foods, like ungulates (Fig. 2A; Appendix 1). If man-eating behaviour would have played a key role in diet, we should expect values elevated in comparison to the local level of human values. The average δ^15^N value of 11 samples of archaeological dentin collected from cementoenamel junction of permanent second premolars of individuals aged around 7–8 years from early nineteenth century Rauma was 11.0‰^44^ (Fig. 1; Appendix 1). Dentine develops during childhood^45^ and its isotope composition reflects the dietary conditions of the growing period^14,15^. On the other hand, food shortages of predators are not only connected to man-eating behaviour^18,46^ but may urge wolves to eat more plant-based nutrition^47^. Even normally, the occurrence of berries in wolfs diet in Finland varies between 1–3%^9^. This could have significantly dragged down the δ^15^N values.

Much like the Tsavo man-eating lion case^18^, the last months of the St. Olaf’s school wolf are well documented, and we have relatively accurate records of the number and age of the children killed and eaten. However, the proportion of the human tissue in the diet of Tsavo lions was up to 30%, clearly more than the 10–15% estimated for the man-eating wolves. Such a large portion is much more likely to show up in the stable isotope composition of the hair keratin and additionally, in the study by Yeakel and colleagues^18^, the possibility to compare the results with values of the bone collagen provided more potential for interpretation.

Ungulates and moose in particular are the main source of nutrition for wolves^8,9^, but in the nineteenth-century, European moose (*Alces alces alces*) population in Finland was markedly reduced^12^. However, we could not detect isotopic signs of starvation, which in inert tissues may be observed as δ^15^N value elevation and sometimes as simultaneous depletion of ^13^C^27–30^. Rather, the δ^13^C and δ^15^N values of both the suspected man-eaters, as well as those of the references group are well in line with a diet possibly containing a wide variety of herbivorous and omnivorous animal species from terrestrial C_3_ plant-based community^10,11^ (Fig. 2A; Appendix 1). As mentioned earlier, in year 1878 alone, 12 pigs, 2455 sheep, 472 cows, and 158 horses were reportedly lost for predators, probably mostly wolves. The δ^15^N and δ^13^C values of the suspected man-eating wolves are well in line with consuming domesticated species (Fig. 2A; Appendix 1). The transition to killing and eating children may have been easy for wolves familiar to proximity of humans via their domestic animals.

The isotopic ratios of nitrogen also suggests inclusion of some aquatic species from brackish or freshwater environments—such as fish-eating waterfowl—as a part of the diets of the wolves particularly in the control group^48^. The δ^15^N values are at a high level in comparison to modern wolf values although even much higher levels have been obtained, e.g. up to 12.9‰ in Alaskan wolves relying much on Pacific salmon^48^. In this geographic region, the δ^13^C values were not helpful in reliably distinguishing between marine and terrestrial/freshwater origin^26,49^ but typically, the tissues of species at high levels of the long and complex aquatic food-webs are enriched in ^15^N^24,50^.

As a main limitation of this study, we can only estimate the amount of human biomass consumed by the St. Olaf’s school wolf. It likely covered less than a tenth of what was nutritionally required by the pack. It is also unlikely that all the human tissue would have been consumed by an individual wolf. As the wolf pack of Turku consisted of an adult alfa pair and their offspring, there were two or three wolves sharing the kill. We can only assume how they distributed their prey. The bone collagen stable isotope results of the St Olaf’s wolf could have provided information on the baseline diet of that individual against which to reflect the keratin analysis results, but unfortunately the analyses were unsuccessful.

The man-eating behavior of carnivores is often connected to problems with their usual diet. This may have been the case also with the man-eating wolves of Turku. The significant reduction of the moose population in the late nineteenth century, the main prey of wolves, may have led some wolves to rely on atypical nutrition. The δ^13^C and δ^15^N values of the suspected man-eaters, align well with a diet that contained a variety of herbivorous and omnivorous animal species from terrestrial C_3_ plant-based community. These likely included domestic animals, but also the man-eating behavior remained as a possibility as it would have formed just a minor part of the total diet and thus could not significantly affect the isotopic values.

## Methods

### Human biomass

Man-eating wolves killed 22 children aged 2–10 years in 1880 and 1881. Although these killings are well documented, assessing the potential amount of human tissue or biomass consumed by the alpha male presents several challenges. Firstly, most of the prey was only partially consumed by these wolves as they were often disturbed while eating, resulting in the incomplete consumption of the body^1^. Secondly, while the age and sex of the victims are known, there may be a significant variation in body size among individuals of the same age. Additionally, the body size of children from the 1880s is not directly comparable to modern-day values. In our estimations, we opted to use modern-day children’s growth charts from Finland, and instead of using the normal growth rate values directly, we subtracted them by two standard deviations.

### Stable isotope analyses

The stable isotope ratios of carbon and nitrogen were analyzed from the keratin of body hair collected from the taxidermized hide of the suspected man-eating alpha male from St. Olaf’s school. Taxidermy has previously been found not to affect the stable isotope values^51^. The hair was analyzed in c. 1 cm sequences to scrutinize any possible temporal change in diet during the growth period of the hair. The minimum length of this hair was 6 cm. An additional seventh sample from the remaining hair tips of varying lengths was analysed, representing the furthest time from the animal’s death. Based on the deduced growth rate of 0.63 mm/day^16^, the isotopic composition of these hairs can be assumed to reflect the dietary conditions of roughly the last 4 months of the wolf’s life, approximately the period beginning from September.

Additionally, an attempt was made to collect a bone sample from the mandible of the taxidermized St. Olaf’s school wolf. The bone collagen extraction was performed using suction filtering according to a modified Longin method introduced by Brown and colleagues^52^ and analysed in the same patch with the hair samples.

In addition, we analysed two wolves possibly involved in man-eating but with much less documented history than the St. Olaf’s school wolf. These samples were a taxidermized wolf 359 from Turku-region (late nineteenth century; the collections of Lammi Biological station, University of Helsinki) and a wolf hide originally from Turku region (late nineteenth century; the collections of the Forssa Nature Museum). The exact dates of the hunting of these individuals are missing, but wolf 359 is marked as “man-eater” in the collection, and the hide from the Forssa Nature Museum is also suspected to have potentially originated from a wolf belonging to a man-eating pack. The hair sample of wolf 359 was obtained from the mid-shafts of the hair while the Forssa sample was complete hairs due to their shortness, and thus they represent the diet some months prior to the death.

Nevertheless, rather than merely the most recent diet, stable isotope signals also convey the composition of the organism’s former amino acid pool, the primary source for protein synthesis. This buffering effect mitigate immediate dietary changes from fully manifesting in tissues, although some nitrogen atoms from the new diet will gradually begin to incorporate among the old ones, altering the composition of growing tissues^20^. Therefore, even the earlier human casualties may be relevant in this case.

The reference group of concurrent wolves consists of samples taken from six wolf hides belonging to the collections of The Hunting Museum of Finland. The provenance of these subjects is uncertain but they all date to the nineteenth century and at least four of them were shot by Anders Edvard Baskin presumably in 1860–1880, in Kymi Valley. One taxidermized wolf 400 (coll. 29.9.1845, Pornainen) was from the collections of Lammi Biological station, University of Helsinki. The reference samples from the control group were obtained from the mid-shafts of the hair, and they represent the diet some months prior to the death.

The wolf hair samples were prepared for the carbon and nitrogen stable isotope analyses at the Archaeological Research Laboratory, Stockholm University, Stockholm, Sweden, with the exception of the Forssa Nature Museum sample, which was prepared in the Nuclear Research Department, Center for Physical Sciences and Technology, Vilnius, Lithuania. To remove lipids, they were ultrasonicated for 3 × 15 min in a chloroform–methanol solution (2:1) following the modified protocol of Britton and colleagues^53^. Subsequently, the hair was rinsed of any residue by ultrasonically cleaning them for 1 × 15 min in ultrapure water and left to dry for 24 h, before being cut and weighed into tin cups in samples ranging from 0.7 to 1.1 mg.

The isotope analyses were conducted at the Nuclear Research Department, Center for Physical Sciences and Technology, Vilnius, Lithuania. Samples were transferred to the tin capsules and analysed using Flash EA 1112 series Elemental analyzer that was connected to the Delta V Advantage Isotope Ratio Mass Spectrometer (IRMS) via ConFlo III interface (all from Thermo, Bremen, Germany). The Elemental analyzer consists of oxidation and reduction furnaces (operating at 1020 °C and 650 °C, respectively), a chromatographic column (PoraPlot Q), a water absorption column, and a TCD detector. During the analysis, N% and C% were determined using the Elemental analyzer, and later gases were passed to the IRMS for the stable isotope ratio measurements. Ratios of ^15^N/^14^N and ^13^C/^12^C were expressed relative to the international standards, atmospheric air (N) and Pee Dee Belemnite ©, and presented in a δ-notation as parts per thousand difference from the standard. Caffeine IAEA-600 was used as a secondary reference material (δ^15^N = + 1‰, δ^13^C = − 27.771‰); the analytical precision was 0.1‰ for both δ^15^N and δ^13^C.

Statistical analyses, including testing for normal distribution of the δ^15^N and δ^13^C values and comparing the St. Olaf’s school wolf and two other potential man-eaters with the reference material with Mann–Whitney tests, were conducted by using SPSS (IBM, Armonk, NY, USA) version 25, 64-bit edition. P-values were considered statistically significant if they were smaller than 0.05.

We also compared the hair keratin stable isotope values of the studied wolves to bone or dentine collagen stable isotope values of wild and domestic herbivores, domestic omnivores, and birds from the dIANA database (Appendix 1). Additionally, we compared them to the early nineteenth century human population of the town of Rauma located near Turku^44^ (Fig. 1). To make this comparison, keratin values were modified to correspond to the more frequently used collagen values, following the suggestions of O’Connell and Hedges^20^ by adding + 0.5‰ to the δ^13^C values and 1‰ to the δ15N values. Additionally, the δ^13^C values were adjusted for the SUESS effect according to the table presented by McCarroll and Loader^54^.

### Supplementary Information

Supplementary Information.

## Data Availability

All data is available upon reasonable request from the corresponding author.
