# Supplementary material for: Stable isotope analyses of carbon and nitrogen in hair keratin of suspected man-eating wolves from 1880s
Source: Sci Rep. 2024 Feb 28;14:4946. doi: 10.1038/s41598-024-55521-8 (PMC10902326; doi:10.1038/s41598-024-55521-8)
Supplement: Supplementary file 1 — Supplementary Information. [file 41598_2024_55521_MOESM1_ESM.docx]

*Appendix 1. The faunal background bone/dentin collagen isotopic data collected from dIANA database [49] and human dentin values from early 19^th^ century population of Rauma [44]. The same values are presented in the Figure 2.A.*

| **Species** | **Site** | **δ^13^C** | **δ^15^N** | **δ^13^C mod.** | **C%** | **N%** | **C/N atomic** | **Yield %** | **Reference** |
| --- | --- | --- | --- | --- | --- | --- | --- | --- | --- |
| *Alces alces* | Helsinki zoo | -21.0 | 5.9 | -20.7 | 42.7 | 15.4 | 3.2 |  | Etu-Sihvola et al. 2019 |
| *Alces alces* | Helsinki zoo | -22.8 | 1.1 | -22.4 | 42.0 | 14.6 | 3.4 |  | Etu-Sihvola et al. 2019 |
| *Alces alces* | Helsinki zoo | -21.2 | 5.5 | -20.9 | 43.8 | 15.8 | 3.2 |  | Etu-Sihvola et al. 2019 |
| *Alces alces* | Nurmes | -21.8 | 1.9 | -21.5 | 43.3 | 15.6 | 3.2 |  | Etu-Sihvola et al. 2019 |
| *Bos taurus* | Vesilahti | -22.4 | 4.9 | -22.0 | 38.6 | 13.3 | 3.4 |  | Bläuer et al. 2016 |
| *Bos taurus* | Pihtipudas | -21.6 | 4.2 |  | 41.9 | 15.2 | 3.2 |  | Bläuer et al. 2016 |
| *Bos taurus* | Naantali | -21.8 | 4.2 |  | 41.2 | 14.5 | 3.3 |  | Bläuer et al. 2016 |
| *Bos taurus* | Parikkala | -22.0 | 3.3 |  | 42.4 | 15.2 | 3.3 |  | Bläuer et al. 2016 |
| *Bos taurus* | Parikkala | -21.7 | 2.9 |  | 42.2 | 14.6 | 3.4 |  | Bläuer et al. 2016 |
| *Bos taurus* | Loviisa | -22.1 | 4.6 |  | 43.1 | 15.3 | 3.3 |  | Bläuer et al. 2016 |
| *Bos taurus* | Kökar | -21.6 | 5.1 |  | 43.0 | 15.4 | 3.3 |  | Bläuer et al. 2016 |
| *Bos taurus* | Nakkila | -22.5 | 5.9 |  | 42.9 | 14.9 | 3.4 |  | Bläuer et al. 2016 |
| *Bos taurus* | Oulu | -23.1 | 2.8 |  | 40.6 | 14.1 | 3.4 |  | Lahtinen & Salmi 2018 |
| *Bos taurus* | Oulu | -22.3 | 4.8 |  | 43.1 | 14.9 | 3.4 |  | Lahtinen & Salmi 2018 |
| *Bos taurus* | Isokyrö | -22.6 | 6.4 |  | 40.3 | 13.0 | 3.6 |  | Oinonen et al. 2020 |
| *Bos taurus* | Isokyrö | -21.9 | 4.5 | -21.5 | 42.6 | 15.0 | 3.3 |  | Oinonen et al. 2020 |
| *Bos taurus* | Isokyrö | -21.6 | 5.6 |  | 40.6 | 14.6 | 3.3 |  | Oinonen et al. 2020 |
| *Canis familiaris* | Isokyrö | -19.5 | 11.0 |  | 41.2 | 14.8 | 3.3 |  | Oinonen et al. 2020 |
| *Castor fiber* | Isokyrö | -22.1 | 2.0 | -21.8 | 42.2 | 15.1 | 3.2 |  | Etu-Sihvola et al. 2019 |
| *Equus caballus* | Isokyrö | -22.7 | 5.5 |  | 39.6 | 14.0 | 3.3 |  | Oinonen et al. 2020 |
| *Equus caballus* | Isokyrö | -22.1 | 7.8 | -21.9 | 38.9 | 14.0 | 3.2 |  | Oinonen et al. 2020 |
| *Equus caballus* | Isokyrö | -22.6 | 5.7 |  | 38.5 | 13.9 | 3.2 |  | Oinonen et al. 2020 |
| *Lepus timidus* | Oulu | -22.4 | 1.6 |  | 44.6 | 14.9 | 3.5 |  | Lahtinen & Salmi 2018 |
| *Lepus timidus* | Oulu | -22.8 | 3.0 |  | 42.2 | 15.3 | 3.2 |  | Lahtinen & Salmi 2018 |
| *Lepus timidus* | Oulu | -23.4 | 1.3 | -23.1 | 45.2 | 16.0 | 3.3 |  | Etu-Sihvola et al. 2019 |
| *Lepus timidus* | Halikko | -24.2 | 4.2 | -23.9 | 44.5 | 15.2 | 3.4 |  | Etu-Sihvola et al. 2019 |
| *Lepus timidus* | Elimäki | -23.7 | 3.1 | -23.4 | 48.2 | 16.9 | 3.3 |  | Etu-Sihvola et al. 2019 |
| *Lepus timidus* | Kirkkonummi | -23.4 | 2.6 | -23.1 | 44.0 | 15.3 | 3.4 |  | Etu-Sihvola et al. 2019 |
| *Lepus timidus* | Vehkalahti | -23.8 | 2.6 | -23.4 | 43.1 | 15.1 | 3.3 |  | Etu-Sihvola et al. 2019 |
| Ovis/Capra | Halikko | -21.6 | 3.1 |  | 43.6 | 15.0 | 3.4 |  | Bläuer et al. 2016 |
| Ovis/Capra | Parikkala | -21.9 | 5.1 |  | 40.8 | 14.5 | 3.3 |  | Bläuer et al. 2016 |
| Ovis/Capra | Naantali | -21.6 | 6.6 | -21.6 | 41.4 | 14.4 | 3.4 |  | Bläuer et al. 2016 |
| Ovis/Capra | Turku | -21.8 | 7.0 |  | 42.2 | 14.7 | 3.3 |  | Bläuer et al. 2016 |
| Ovis/Capra | Loviisa | -21.4 | 5.8 |  | 34.8 | 12.7 | 3.2 |  | Bläuer et al. 2016 |
| Ovis/Capra | Kökar | -22.1 | 7.3 |  | 41.5 | 15.0 | 3.2 |  | Bläuer et al. 2016 |
| Ovis/Capra | Kökar | -20.8 | 5.2 |  | 40.1 | 14.8 | 3.1 |  | Bläuer et al. 2016 |
| Ovis/Capra | Isokyrö | -21.9 | 6.0 | -21.8 | 40.5 | 14.4 | 3.3 |  | Oinonen et al. 2020 |
| Ovis/Capra | Oulu | -22.0 | 5.0 |  | 44.0 | 15.6 | 3.3 |  | Lahtinen & Salmi 2018 |
| Ovis/Capra | Oulu | -21.7 | 7.4 |  | 42.9 | 14.8 | 3.4 |  | Lahtinen & Salmi 2018 |
| *Pusa hispida botniensis* | Lapua | -19.3 | 13.6 | -18.9 | 45.3 | 16.0 | 3.3 |  | Etu-Sihvola et al. 2019 |
| *Pusa hispida botniensis* | Kökar | -15.7 | 13.8 | -15.3 | 43.2 | 15.2 | 3.3 |  | Etu-Sihvola et al. 2019 |
| *Pusa hispida botniensis* | Nurmo | -19.5 | 13.8 | -19.1 | 41.4 | 14.2 | 3.4 |  | Etu-Sihvola et al. 2019 |
| *Pusa hispida botniensis* | Vaasa | -16.5 | 12.7 | -16.2 | 42.6 | 15.1 | 3.3 |  | Etu-Sihvola et al. 2019 |
| *Pusa hispida botniensis* | Utö | -16.9 | 13.0 | -16.6 | 43.1 | 14.6 | 3.5 |  | Etu-Sihvola et al. 2019 |
| Rangifer | Oulu | -19.6 | 5.5 |  | 43.0 | 14.9 | 3.4 |  | Lahtinen & Salmi 2018 |
| Rangifer | Rovaniemi | -20.1 | 3.9 |  | 44.9 | 14.8 | 3.5 |  | Lahtinen & Salmi 2018 |
| Rangifer | Tornio | -19.0 | 4.1 |  | 44.6 | 15.6 | 3.3 |  | Lahtinen & Salmi 2018 |
| Rangifer | Ylikylä | -19.5 | 5.6 |  | 41.3 | 16.0 | 3.1 |  | Salmi & Heino 2019 |
| Rangifer | Ylikylä | -19.1 | 5.4 |  | 41.4 | 15.0 | 3.2 |  | Salmi & Heino 2019 |
| Rangifer | Oravaisensaari | -19.7 | 2.7 |  | 39.2 | 15.0 | 3.2 |  | Salmi & Heino 2019 |
| Rangifer | Oravaisensaari | -18.5 | 2.0 |  | 35.8 | 13.0 | 3.1 |  | Salmi & Heino 2019 |
| Rangifer | Hyrynsalmi | -20.7 | 3.0 | -20.3 | 45.0 | 15.4 | 3.4 |  | Etu-Sihvola et al. 2019 |
| *Sus domesticus* | Oulu | -22.3 | 5.5 |  | 43.5 | 15.0 | 3.4 |  | Lahtinen & Salmi 2018 |
| *Sus domesticus* | Oulu | -21.7 | 9.9 |  | 43.5 | 14.6 | 3.5 |  | Lahtinen & Salmi 2018 |
| Tetraoninae | Salla | -21.1 | 1.8 | -20.7 | 46.1 | 16.3 | 3.3 |  | Etu-Sihvola et al. 2019 |
| Tetraoninae | Inari | -21.1 | 0.5 | -20.8 | 43.6 | 15.4 | 3.3 |  | Etu-Sihvola et al. 2019 |
| Tetraoninae | Helsinki zoo | -21.8 | 3.7 | -21.4 | 42.8 | 15.0 | 3.3 |  | Etu-Sihvola et al. 2019 |
| Tetraoninae | Muonio | -20.2 | 1.7 | -20.1 | 46.0 | 15.9 | 3.4 |  | Etu-Sihvola et al. 2019 |
| Tetraoninae | Tampere | -22.5 | 2.7 | -22.2 | 45.3 | 15.7 | 3.4 |  | Etu-Sihvola et al. 2019 |
| Tetraoninae | Oulu | -21.6 | 2.3 |  | 44.0 | 14.5 | 3.5 |  | Lahtinen & Salmi 2018 |
| Tetraoninae | Oulu | -22.4 | 1.7 |  | 42.1 | 14.4 | 3.4 |  | Lahtinen & Salmi 2018 |
| Tetraoninae | Kuusamo/Helsinki zoo | -21.9 | 4.0 | -21.5 | 43.5 | 15.3 | 3.3 |  | Etu-Sihvola et al. 2019 |
| Tetraoninae | Helsinki zoo | -21.3 | 7.7 | -21.0 | 43.0 | 15.1 | 3.3 |  | Etu-Sihvola et al. 2019 |
| Tetraoninae | Kuusamo/Helsinki zoo | -21.9 | 4.2 | -21.5 | 42.5 | 15.2 | 3.3 |  | Etu-Sihvola et al. 2019 |
| Tetraoninae | Kittilä/Sodankylä | -21.9 | 2.1 | -21.6 | 43.7 | 15.4 | 3.3 |  | Etu-Sihvola et al. 2019 |
| *Ursus arctos* | Kajaani | -19.8 | 8.5 | -19.5 | 46.5 | 16.2 | 3.4 |  | Etu-Sihvola et al. 2019 |
| *Ursus arctos* | Helsinki zoo | -19.6 | 5.0 | -19.3 | 43.7 | 15.6 | 3.3 |  | Etu-Sihvola et al. 2019 |
| *Ursus arctos* | Inari | -19.0 | 5.8 | -18.7 | 42.3 | 14.8 | 3.3 |  | Etu-Sihvola et al. 2019 |
| *Anas penelope* | Helsinki | -22.1 | 7.8 | -21.7 | 43.8 | 14.9 | 3.4 |  | Etu-Sihvola et al. 2019 |
| *Anas* species | Oulu | -23.4 | 8.2 |  | 41.9 | 14.9 | 3.3 |  | Lahtinen & Salmi 2018 |
| *Anser anser* | Helsinki zoo | -22.4 | 8.3 | -22.1 | 44.1 | 14.8 | 3.5 |  | Etu-Sihvola et al. 2019 |
| *Anser anser* | Helsinki zoo | -20.8 | 8.7 | -20.4 | 44.8 | 15.4 | 3.4 |  | Etu-Sihvola et al. 2019 |
| *Anser anser* | Helsinki | -24.0 | 9.8 | -23.6 | 46.5 | 17.4 | 3.1 |  | Etu-Sihvola et al. 2019 |
| *Anser fabalis* | Perho | -22.8 | 6.4 | -22.5 | 42.0 | 14.4 | 3.4 |  | Etu-Sihvola et al. 2019 |
| *Anser fabalis* | Helsinki zoo | -21.7 | 9.5 | -21.4 | 43.8 | 15.5 | 3.3 |  | Etu-Sihvola et al. 2019 |
| *Anser fabalis* | Helsinki zoo | -22.6 | 7.3 | -22.3 | 39.9 | 13.4 | 3.5 |  | Etu-Sihvola et al. 2019 |
| Human FDI5 dentin 12 | Rauma | -20.1 | 11.4 |  | 38.26 | 14.34 | 3.1 | 3.5 | Väre et al. 2022 |
| Human FDI5 dentin 122 | Rauma | -20.3 | 11.5 |  | 38.76 | 14.27 | 3.2 | 5.5 | Väre et al. 2022 |
| Human FDI5 dentin 123 | Rauma | -19.7 | 10.9 |  | 38.36 | 14.53 | 3.1 | 5.9 | Väre et al. 2022 |
| Human FDI5 dentin 124 | Rauma | -19.8 | 11.4 |  | 38.06 | 13.95 | 3.2 | 3.3 | Väre et al. 2022 |
| Human FDI5 dentin 150 | Rauma | -19.8 | 11.7 |  | 37.87 | 13.84 | 3.2 | 4.7 | Väre et al. 2022 |
| Human FDI5 dentin 151 | Rauma | -20.4 | 10.5 |  | 38.99 | 14.32 | 3.2 | 4.2 | Väre et al. 2022 |
| Human FDI5 dentin 166 | Rauma | -19.8 | 11.6 |  | 39.95 | 14.62 | 3.2 | 7.5 | Väre et al. 2022 |
| Human FDI5 dentin 196 | Rauma | -20.5 | 9.9 |  | 37.59 | 14.46 | 3.0 | 5.7 | Väre et al. 2022 |
| Human FDI5 dentin 197 | Rauma | -20.7 | 11.5 |  | 39.94 | 14.59 | 3.2 | 6.7 | Väre et al. 2022 |
| Human FDI5 dentin 200 | Rauma | -20.7 | 10.4 |  | 38.49 | 13.94 | 3.2 | 7.0 | Väre et al. 2022 |
| Human FDI5 dentin 208 | Rauma | -19.9 | 10.5 |  | 36.83 | 14.93 | 2.9 | 4.9 | Väre et al. 2022 |
